# Supplementary material for: The supernova remnant SN 1006 as a Galactic particle accelerator
Source: Nat Commun. 2022 Aug 30;13:5098. doi: 10.1038/s41467-022-32781-4 (PMC9427765; doi:10.1038/s41467-022-32781-4)
Supplement: Supplementary file 2 — Description of Additional Supplementary Files [file 41467_2022_32781_MOESM2_ESM.docx]

**Description of Additional Supplementary Files**

**File Name: Supplementary Software 1
Description:** Supplementary Python code to compute the volume of the X-ray emitting plasma.
